# Optimization of Location–Routing Problem for Cold Chain Logistics Considering Carbon Footprint

**DOI:** 10.3390/ijerph15010086

**Published:** 2018-01-06

**Authors:** Songyi Wang, Fengming Tao, Yuhe Shi

**Affiliations:** 1 College of Mechanical Engineering, Chongqing University, Chongqing 400044, China; songyi_wang@cqu.edu.cn; 2 School of Economics and Business Administration, Chongqing University, Chongqing 400044, China; 3 School of Transportation and Logistics, Southwest Jiaotong University, Chengdu 610031, China; SHI681242@163.com

**Keywords:** cold chain logistics, location-routing problem, hybrid genetic algorithm, carbon emission

## Abstract

In order to solve the optimization problem of logistics distribution system for fresh food, this paper provides a low-carbon and environmental protection point of view, based on the characteristics of perishable products, and combines with the overall optimization idea of cold chain logistics distribution network, where the green and low-carbon location–routing problem (LRP) model in cold chain logistics is developed with the minimum total costs as the objective function, which includes carbon emission costs. A hybrid genetic algorithm with heuristic rules is designed to solve the model, and an example is used to verify the effectiveness of the algorithm. Furthermore, the simulation results obtained by a practical numerical example show the applicability of the model while provide green and environmentally friendly location-distribution schemes for the cold chain logistics enterprise. Finally, carbon tax policies are introduced to analyze the impact of carbon tax on the total costs and carbon emissions, which proves that carbon tax policy can effectively reduce carbon dioxide emissions in cold chain logistics network.

## 1. Introduction

Green and healthy products have gradually become the primary standard for consumers in choosing fresh foods [1]. The shelf life of fresh agricultural products is relatively short; in order to reduce product decay and ensure the health of consumers, it should always be processed, packaged, and transported at suitable temperatures and delivered to consumers in the shortest possible time, which has promoted the rapid development of cold chain logistics to a certain extent. The development of cold chain logistics has led to rapid economic development but poses a series of threats to the environment. Compared with conventional logistics, the normal operation of refrigeration equipment produces a large amount of carbon emissions in cold chain logistics, coupled with CO_2_ emissions and other gases generated from distribution vehicles in the process of exercising, which will lead to an increase of greenhouse gases and, as a result, air pollution and greenhouse effect will be intensified. Therefore, how to reduce carbon emissions in cold chain logistics, and thus alleviate the global warming caused by the greenhouse effect, has become a hot issue in the current research. 

**Figure 1:**
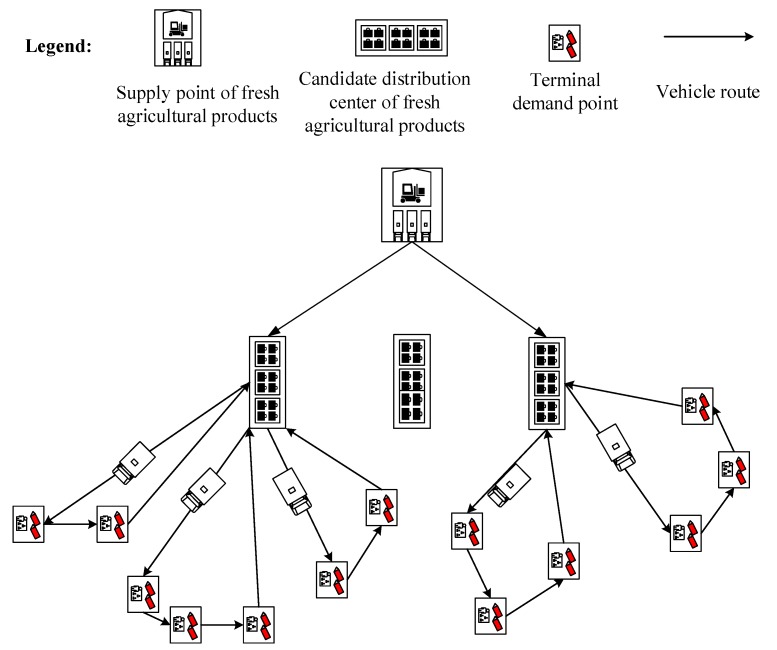
A simplified diagram of the cold chain logistics network.

It is necessary to optimize the design of the cold chain logistics network while considering the benefits and the environment constraints, thus attaining the balance of economic and environmental benefits and then achieving a win-win situation. What the optimal design of cold chain logistics network should take into account is to transport products from the supply points (SPs) to distribution centers (DCs), and then transfer products from DCs to each terminal demand point (TDP) under the constraints of limited time, space, resources, and others. A simplified diagram of the cold chain logistics network is shown in Figure 1. Thus the Location Allocation Problem (LAP) of cold chain logistics network node and Vehicle Routing Problem (VRP) are two key and among the most challenging issues in the optimization design of cold chain logistics network [2]. Different LAP schemes lead to different VRP planning in cold chain logistics distribution, and VRP planning results in turn will affect the LAP schemes [3]. As a result, solving the joint decision problem of LAP and VRP in distribution, that is, the Location–Routing Problem (LRP) in cold chain logistics network, is essential [4].

The remaining parts of this paper are organized as follows. Section 2 introduces a comprehensive literature review on the LRP in the cold chain logistics. Section 3 discusses the construction of the low-carbon LRP (LCLRP) model. Section 4 describes the hybrid genetic algorithm to solve the model. A numerical example is given in Section 5. Finally, Section 6 concludes with some discussions.

## 2. Literature Review

Since joint optimization of the location problem and routing problem is first proposed by Boventer in 1961 [5], LRP gradually became a research hotspot in the field of logistics optimization, and many variants are derived, such as dynamic LRP [3], multi-layer LRP [6], multi-target LRP [2,7,8], and so on. However, there are few researches on the application of LRP in cold chain logistics. On the contrary, the studies of LAP and VRP in the cold chain logistics have made abundant achievements.

About the research of distribution center location in cold chain logistics, Marija Bogataj [9] considered the distribution center as an intermediate link of the circulation, which determined the operational efficiency of the cold chain, in order to ensure the quality of fresh food in cold chain. The location problem of distribution center was studied on the basis of the overall analysis of cold chain. Ioannis Manikas [10] analyzed the main problems in the logistics and distribution of fresh products and pointed out that distribution centers play a key role in the distribution efficiency. Therefore, attention should be given to the location of distribution centers, so as to promote the improvement of the operating performance of distribution centers in the fresh agricultural products distribution industry in the United Kingdom. The location of perishable food distribution centers was studied by Zvi Drezner [11], who suggested that the location of the distribution center would affect the inventory costs and location costs, so the two costs were considered in the total cost calculation. Morteza Zangeneh [12] proposed that service, cost, and speed were important factors influencing the distribution of fresh agricultural products, then the indicators that affect the location of agricultural service centers were identified, and the weights of these factors were calculated by fuzzy analytic hierarchy process.

About the research of distribution vehicle routing optimization in cold chain logistics, Osvald A et al. [13] constructed a routing optimization model of vegetable distribution based on the consideration of the impact of vegetable perishability, driving time, and delivery time on distribution costs, and a tabu search optimization algorithm was designed to solve the model. In order to achieve the purpose of improving the transportation efficiency of agricultural products and customer satisfaction, a linear programming method of operations research was adopted by Alio and Van Oudheusden D [14] to optimize the distribution route of logistics transportation. Chen et al. [15] studied the production of fresh products and its logistics distribution problem with transportation time window, where the objective function of the mathematical model aimed to maximize supplier profit. Based on the predicted parameters of the cold chain product spoilage, Miroslav [16] analyzed the spoilage process of perishable products in the cold chain distribution and formulated the function according to the change process of spoilage degree with time, thus a cold chain distribution vehicle routing optimization model considering spoilage time variation was proposed. Monitanari [17] used two classical mathematical algorithms to solve the cold chain logistics delivery sequence planning problem, which improved the scientific nature of the model. Wee Kit [18] combined the genetic algorithm and tabu search algorithm to solve the cold chain logistics distribution vehicle routing planning model, so that the algorithm had better ability to search for better optimal solution.

In the rare cold chain logistics LRP researches, Li Kang et al. [19] proposed a novel location inventory routing model to optimize costs in cold logistics under uncertain demand environment. A new discrete particle swarm optimization is introduced to solve the integrated model. Zheng Guohua et al. [20] proposed an optimal location–routing model considering temperature variation among sensitive products for cold chain distribution centers, and, as a solution method, a hybrid genetic algorithm-tabu search was proposed. Shi Zhao and Fu Zhuo [21] designed a satisfaction degree function according to service time windows and established the simulation model under time-dependent, and introduced the minimum envelope clustering analysis method and tabu search algorithm to solve the problem.

In short, based on the above analysis, there are many researches on cold chain logistics LAP and VRP. However, there are few LRP studies about fresh agricultural products. Whether it is a basic LRP [22] or a variant of the LRP [23], most of them only take the operation cost as the optimization target and seldom consider the carbon emissions. However, with the concept of sustainable development gradually gaining popular support, a number of energy-saving and emission reduction policies are being implemented, and carbon emission in the operation of cold chain logistics distribution has become a key problem for logistics companies to solve. In view of this, this paper proposes a green and environmental protection model that considers carbon emissions in the optimization of cold chain logistics networks: the low-carbon location–routing problem (LCLRP) model. The hybrid genetic algorithm is used to solve the problem. Finally, the validity and feasibility of the model are verified by a numerical experiment.

## 3. Model Formulation

### 3.1. Problem Description

The LCLRP model of cold chain logistics in this paper can be described as follows. There are multiple cold chain logistics candidate distribution centers delivering products to different customer by using refrigerated vehicles, and the customer locations are known. A refrigerated vehicle starts from a distribution center and will return to the nearest distribution center after completing the delivery. Under the restrictions of customer demand and vehicle capacity, the LCLRP model with the lowest comprehensive costs is constructed by considering the fixed costs, transportation costs, refrigeration costs, penalty costs, damage costs, and carbon emission costs, thus obtaining economic and environmental protection location-distribution scheme and ensuring the completion of distribution services simultaneously.

### 3.2. Parameters and Variables

According to the needs to build the model, this paper sets the following parameters and variables, as shown in Table 1.

### 3.3. Model Development

The cold chain logistics LCLRP model constructed in this paper takes the total costs minimum as the objective function. Firstly, the sub-costs should be analyzed, and then the total costs of the location-distribution process are determined by the various sub-costs.

#### 3.3.1. Objective Function Analysis of Model

1. Fixed Costs

The fixed costs refer to the operation costs of the distribution center, which mainly includes the daily maintenance and depreciation costs of warehouses and vehicles, as well as the labor costs of drivers and other employees [24]. The fixed costs $C_{1}$ in the LCLRP model can be expressed as:

(1) $$C_{1}=\sum_{g\in L_{g}}C_{g}Z_{g}+\sum_{g\in L_{g}}\sum_{k\in K_{g}}Z_{g}Y_{k}C_{k}.$$

2. Transportation Costs

The transportation costs of refrigerated trucks are only considered in this section; the refrigeration costs during transportation will be analyzed separately. The transportation costs of the vehicles are affected by fuel consumption, maintenance, and other factors, and it is proportional to the mileage traveled by the vehicles [25]. The transportation costs $C_{2}$ in the LCLRP model can be expressed as:

(2) $$C_{2}=\sum_{g\in L_{g}}\sum_{k\in K_{g}}\sum_{i,j\in V_{g}}c_{ij}^{k}x_{ij}^{k}d_{ij}Z_{g}Y_{k}.$$

3. Refrigeration Costs

Perishability is one of the characteristics of cold chain logistics products [26]. Therefore, the cold chain logistics requires that cargoes in all aspects of logistics are always in a low-temperature environment to ensure their quality. Energy must be constantly consumed to maintain the same temperature in the process of distribution. This refrigeration cost is necessary in order to maintain the appropriate temperature.

The refrigeration costs of refrigerated trucks during transportation $C_{31}$ as follows:

(3) $$C_{31}=\sum_{g\in L_{g}}\sum_{k\in K_{g}}\sum_{i\in V_{g}}\sum_{j\in V_{g}}Z_{g}C_{e}x_{ij}^{k}{\hat{t}}_{ij}^{k}.$$

After reaching the TDPs, the refrigeration costs of refrigerated trucks generated from the unloading process $C_{32}$ as follows:

(4) $$C_{32}=\sum_{g\in L_{g}}\sum_{k\in K_{g}}\sum_{j\in V_{g}}Z_{g}C_{e}^{\prime }y_{j}^{k}w_{j}.$$

Thus, the total refrigeration costs $C_{3}$ can be expressed as:

(5) $$C_{3}=C_{31}+C_{32}=\sum_{g\in L_{g}}\sum_{k\in K_{g}}\sum_{i\in V_{g}}\sum_{j\in V_{g}}Z_{g}\left(C_{e}x_{ij}^{k}{\hat{t}}_{ij}^{k}+C_{e}^{\prime }y_{j}^{k}w_{j}\right).$$

where ${\hat{t}}_{ij}^{k}=t_{ij}^{k}+\max\left\{ET_{i}-t_{j}^{k},0\right\}$, $\max\left\{ET_{i}-t_{j}^{k},0\right\}$ indicates the waiting time for the vehicle *k* to service the customer *j* without unloading.

4. Penalty Costs

In the cold chain logistics research, urban traffic congestion has brought great difficulties to the logistics distribution: if the cargoes cannot be sent within the time required by the customer, then a certain penalty costs $C_{4}$ must be paid, expressed as:

(6) $$C_{4}=\sum_{g\in L_{g}}\sum_{k\in K_{g}}\sum_{i\in V_{g}}Z_{g}\left(\mu_{1}\max\left\{ET_{i}-t_{i}^{k},0\right\}+\mu_{2}\max\left\{t_{i}^{k}-LT_{i},0\right\}\right).$$

where $\max\left\{ET_{i}-t_{j}^{k},0\right\}$ indicates the advance arrival time for refrigerated truck *k* service customer *i*; and $\max\left\{t_{i}^{k}-ET_{i},0\right\}$ indicates the late time for refrigerated truck *k* service customer *i*.

5. Damage Costs

The variable function of refrigerated goods quality is introduced in this paper [27]: $D\left(t\right)=D_{0}e^{-\partial t}$, which is used to calculate the damage costs. The damage costs of cargoes are divided into two parts in the process of distribution, including the costs of accumulated cargo damage with time caused by refrigerated trucks in the travel process, and the costs of loss of cargo near the door due to the opening of the door when serving the customer.

The cargoes damage costs caused by refrigerated trucks in the travel process $C_{51}$ can be defined as:

(7) $$C_{51}=\sum_{g\in L_{g}}\sum_{k\in K_{g}}\sum_{i\in V_{g}}Z_{g}y_{i}^{k}Pq_{i}\left(1-e^{-\partial_{1}\left(t_{i}^{k}-t_{0}^{k}\right)}\right).$$

When refrigerated trucks arrive at the customers’ location for service, the spoilage rate is assumed as $\partial_{2}\left(\partial_{2}>\partial_{1}\right)$ at this time, and the costs of cargoes loss $C_{52}$ can be defined as:

(8) $$C_{52}=\sum_{g\in L_{g}}\sum_{k\in K_{g}}\sum_{i\in V_{g}}Z_{g}y_{i}^{k}PQ_{ij}^{k}\left(1-e^{-\partial_{2}t_{si}}\right).$$

Thus, total damage costs as follows:

(9) $$C_{5}=C_{51}+C_{52}=\sum_{g\in L_{g}}\sum_{k\in K_{g}}\sum_{i\in V_{g}}Z_{g}y_{i}^{k}P\left[q_{i}\left(1-e^{-\partial_{1}\left(t_{i}^{k}-t_{0}^{k}\right)}\right)+Q_{ij}^{k}\left(1-e^{-\partial_{2}t_{si}}\right)\right].$$

6. Carbon Emission Costs

In the process of distribution and transportation, carbon emissions are generated from two sources: transportation fuel consumption and equipment refrigeration. In this paper, we introduce the linear function formula of fuel consumption in per unit distance ρ(X) [28,29]:

(10) $$\rho \left(X\right)=\rho_{0}+\frac{\rho^{*}-\rho_{0}}{Q}X.$$

Therefore, the carbon emissions in the transportation process expressed as follows:

(11) $$EM=e_{0}\rho \left(Q_{ij}^{k}\right)d_{ij}+\omega Q_{ij}^{k}d_{ij},(0\leq Q_{ij}^{k}\leq Q_{k}).$$

The carbon emission costs of the LCLRP model include the fixed carbon emissions of the distribution center and the carbon emissions from the distribution and transportation:

(12) $$C_{6}=C_{0}\left[\sum_{g\in L_{g}}C_{g}Z_{g}+\sum_{g\in L_{g}}\sum_{k\in K_{g}}\sum_{i\in V_{g}}\sum_{j\in V_{g}}Z_{g}x_{ij}^{k}d_{ij}\left[e_{0}\rho \left(Q_{ij}^{k}\right)+\omega Q_{ij}^{k}\right]\right].$$

#### 3.3.2. LCLRP Model Setting

Based on the analysis of sub-costs in Section 3.3.1, the LCLRP model of cold chain logistics constructed in this paper is as follows:

(13) $$\begin{array}{cc} \min C & =\left(\sum_{g\in L_{g}}C_{g}Z_{g}+\sum_{g\in L_{g}}\sum_{k\in K_{g}}Z_{g}Y_{k}C_{k}\right)+\left(\sum_{g\in L_{g}}\sum_{k\in K_{g}}\sum_{i,j\in V_{g}}c_{ij}^{k}x_{ij}^{k}d_{ij}Z_{g}Y_{k}\right)\\ & +\left(\sum_{g\in L_{g}}\sum_{k\in K_{g}}\sum_{i\in V_{g}}\sum_{j\in V_{g}}Z_{g}\left(C_{e}x_{ij}^{k}{\hat{t}}_{ij}^{k}+C_{e}^{\prime }y_{j}^{k}w_{j}\right)\right)\\ & +\left(\sum_{g\in L_{g}}\sum_{k\in K_{g}}\sum_{i\in V_{g}}Z_{g}\left(\mu_{1}\max\left\{ET_{i}-t_{i}^{k},0\right\}+\mu_{2}\max\left\{t_{i}^{k}-LT_{i},0\right\}\right)\right)\\ & +\left(\sum_{g\in L_{g}}\sum_{k\in K_{g}}\sum_{i\in V_{g}}Z_{g}y_{i}^{k}P\left[q_{i}\left(1-e^{-\partial_{1}\left(t_{i}^{k}-t_{0}^{k}\right)}\right)+Q_{ij}^{k}\left(1-e^{-\partial_{2}t_{si}}\right)\right]\right)\\ & +C_{0}\left[\sum_{g\in L_{g}}EM_{g}Z_{g}+\sum_{g\in L_{g}}\sum_{k\in K_{g}}\sum_{i\in V_{g}}\sum_{j\in V_{g}}Z_{g}x_{ij}^{k}d_{ij}\left[e_{0}\rho \left(Q_{ij}^{k}\right)+\omega Q_{ij}^{k}\right]\right] \end{array}$$

Subject to

(14) $$\sum_{k\in K_{g}}\sum_{j\in V_{g}}x_{{}_{ij}}^{k}=1,i\in V_{g}$$

(15) $$\sum_{k\in K_{g}}\sum_{j\in V_{g}}x_{{}_{ij}}^{k}=\sum_{k\in K_{g}}\sum_{j\in V_{g}}x_{{}_{ji}}^{k},i\in V_{g}$$

(16) $$\sum_{k\in K_{g}}\sum_{j\in V_{g}}Q_{{}_{ij}}^{k}-\sum_{k\in K_{g}}\sum_{j\in V_{g}}Q_{{}_{ji}}^{k}=q_{i},i\in V_{g}$$

(17) $$\sum_{g\in L_{g}}q_{i}y_{ig}=Q_{g}Z_{g},i\in V_{g}$$

(18) $$\sum_{k\in K_{g}}x_{ig}^{k}\leq Z_{ig},i\in V_{g},g\in L_{g}$$

(19) $$\sum_{k\in K_{g}}x_{ig}^{k}\leq Z_{gi},i\in V_{g},g\in L_{g}$$

(20) $$\sum_{k\in K_{g}}x_{ij}^{k}+\sum_{g_{1}\in L_{g},g_{1}\neq g}x_{ij}^{k}Z_{jg_{1}}+Z_{ig}\leq 2,g\in L_{g},i\in V_{g},j\in V_{g},i\neq j,k\in K_{g}$$

(21) $$Q_{ij}^{k}\leq Q_{k}x_{ij}^{k},i\in V_{g},j\in V_{g},i\neq j,k\in K_{g}$$

(22) $$t_{j}^{k}=t_{i}^{k}+t_{si}+t_{ij}^{k}.$$

The objective function of the model is to minimize the sum of costs, as shown in (13). Constraint (14) represents that there is only one refrigerated vehicle provides delivery service for each customer. Every customer is a service object, as mentioned in (15). Constraint (16) imposes the notion that the demands of each customer will be met. The total customer requirements assigned to the distribution center *g* are not greater than the maximum storage capacity of the distribution center, which is imposed by (17). The refrigerated trucks departing from a distribution center can go to other distribution centers after passing through the demand points that need to be serviced by the distribution center, and its operation is shown in (18)–(20). Constraint (21) indicates that the load of the refrigerated trucks cannot exceed its maximum load. The continuity of the travel time of the refrigerated vehicle is emphasized in (22).

## 4. Algorithm Design

**Figure 2:**
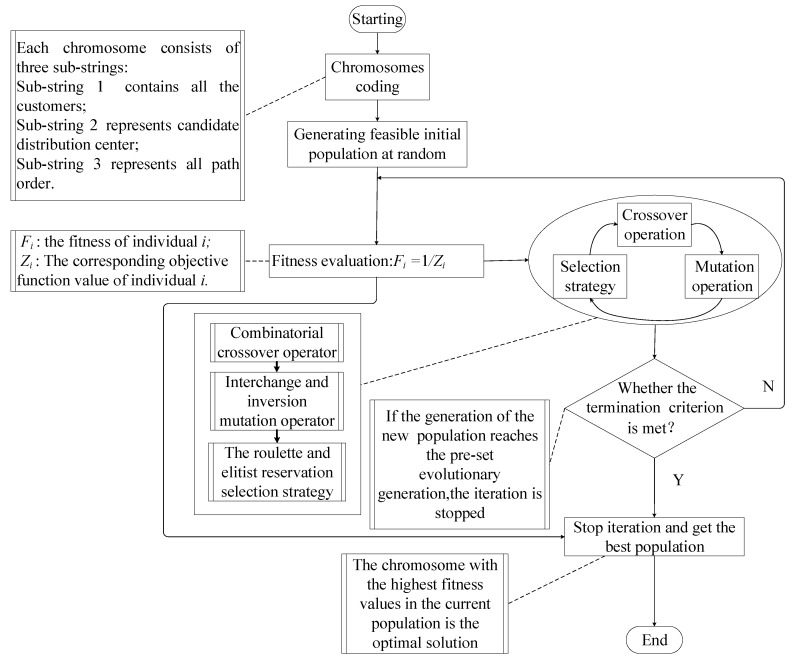
Basic process of hybrid genetic algorithm (HGA).

LRP belongs to the NP-Hard problem [30], and a hybrid genetic algorithm (HGA) combining heuristic rules is designed to solve the model in this paper. Its basic process is illustrated in Figure 2.

Step one: Chromosomes coding. Each chromosome consists of three sub-strings: Sub-string 1 has *n* genes (*n* is the number of TDPs); Sub-string 2 represents the starting distribution center corresponding to each circuit sub-path; Sub-string 3 represents the arrangement order of each demand point in each circuit sub-path. The genes in the sub-string 1 and sub-string 3 correspond to each other, and the demand points in the sub-string 3 corresponding to the genes with the same value in the sub-string 1 are in the same sub-path. In this way, each chromosome contains both a distribution center location-allocation and vehicle routing information.

**Figure 3:**
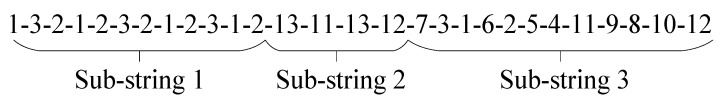
Description of coding example.

For example, there are 12 demand points (numbers 1 to 12) and four candidate distribution centers (numbers 13 to 16), P = 4 (numbers 1 to 4). For the following chromosomes:

As shown in Figure 3, the sub-string 1 indicates that there are three sub-paths. The sub-string 2 indicates that two candidate distribution centers numbered 13 and 11 are established. It should be noted that the fourth gene of sub-string 2 is numbered 12, but the candidate distribution center 12 is not actually established, because there are only three sub-paths in the sub-string 1, and the path numbered 4 is nonexistent. The 1st, 4th, 8th, and 11th genes of sub-string 1 are numbered 1 and the corresponding genes in sub-string 3 are the demand points 7, 6, 11, and 10, which indicates that these demand points are in the sub-path numbered 1 and the driving order in the sub-path 1 is 7-6-11-10. The first gene in sub-string 2 is numbered 13, indicating that the starting point of sub-path 1 is distribution center 13, thus the sub-path 1 is 13-7-6-11-10. Similarly, the sub-path 2 is 11-1-2-4-9-12 and sub-path 3 is 13-3-5-8.

Step two: Initializing the population. The population size is N, and the specific method is as follows: a chromosome is randomly generated and will be retained if the minimum vehicle capacity and distribution center capacity constraints are met; otherwise, another chromosome will be generated, until N chromosomes are generated.

Step three: Population fitness evaluation. The fitness of population was evaluated, $F_{i}=1/Z_{i}$, where $F_{i}$ is the fitness of individual *i*, $Z_{i}$ is the corresponding objective function value of individual *i*. 

Step four: Crossover and mutation operation. In order to maintain the diversity of the population and prevent the generation of error codes, crossover and mutation operations are performed on each sub-string in the chromosome. Single-point crossover and two-point crossover operation are chosen to perform on sub-string 1 and sub-string 2 respectively, and the interchange mutation operation is carried out. Sequence crossover and partial matching crossover operation are used in sub-string 3, and inversion mutation operation is performed. 

Step five: Selection strategy. We choose a selection strategy that combines roulette and elitist reservation for selection operation. When generating the next generation of population, the optimal individuals in the parent population and the temporary population generated by crossover and mutation operations are directly duplicated into the next generation population. The other individuals in the new population are selected from the parent population and the temporary population by roulette.

Step six: Termination condition of algorithm. The maximum iteration number of the genetic algorithm is set as M, and the algorithm terminates when the number of iterations is greater than M, that is, gen > M.

## 5. Experimental Design and Result Analysis

The example validation includes two parts: firstly, the HGA proposed in this paper is tested in Section 5.1 using the internationally accepted Prodhon data set [31,32]; secondly, in Section 5.2, the effectiveness of the LCLRP model is verified by an actual example of the third-party cold chain logistics enterprise.

This paper uses MATLAB R2014a to implement the HGA, and all experiments in this paper are evaluated on PCs with Intel ® Core ™ (Santa Clara, CA, USA) i7-3610QM CPU@ 2.10 GHz 2.10 GHz and memory of 4 GB.

### 5.1. Algorithm Experiment

In this section, the Prodhon data set is used to verify the effectiveness of the proposed HGA. Prodhon designed 30 test data sets for LRP. The test data is named as *n-m-c*, where *n* represents TDPs (the value is 20, 50, 100, and 200, respectively), *m* represents DCs (5 or 10), and *c* represents the geographical location-distribution rule of TDPs. The locations are uniformly distributed when the value of *c* is 1; the locations are randomly distributed when the value of *c* is 2; when the *c* value is 3, it means that half of the locations are evenly distributed and half are randomly distributed.

We choose 10 from 30 test data sets to form HGA test data sets, the traditional genetic algorithm (GA), the cycle evolutionary genetic algorithm (CEGA) [29], and HGA proposed in this paper are used to solve it respectively. Based on the algorithm parameter setting in literature [33,34,35], in this paper, the parameters of HGA are set as follows: the initial populations are 100, the number of evolution iterations is 500, the crossover probability is 0.8, and the mutation probability is 0.2. The results are shown in Table 2 (since the carbon emission costs are not taken into account in the test data sets, for a fair comparison of the performance of the algorithm, here the value of $C_{0}$ is 0). In addition, the *t* in the Table 2 shows the time spent at the end of the algorithm.

As can be seen from Table 2, when solving the above 10 examples, the results obtained by HGA algorithm are 100% better than the results obtained by GA, and nearly 80% are better than the results obtained by CEGA. In terms of computation time, the HGA proposed in this paper is superior to GA and CEGA. Therefore, the HGA is very competitive in solving LRP problems.

### 5.2. Model Experiment

#### 5.2.1. Experimental Design

In this paper, the distribution data of a cold chain Logistics Company is used to verify the LCLRP model. MPF Logistics Company mainly provides warehousing and distribution services for refrigerated foods, such as dairy products, chilled meat, and so on. It has five candidate distribution centers in a certain area with a capacity of 200 tons (the locations of the distribution centers are shown in Table 3). Before arranging a delivery task, a total of 60 orders of demand points are received. The specific information such as the geographical location, time window, and demands are shown in Table 4 (urgently needed goods must be served within the service demand time; ordinary goods may not be served within the demand time, but will acquire a penalty; two kinds of goods can be delivered with a same vehicle, the weight of each goods is 10 kg). All refrigerated vehicles have the same type and their parameters are shown in Table 5. In condition of considering customer needs and urban characteristics, the road transportation is used to deliver goods for the convenience of researching problems. Moreover, all roads are non-forbidden roads. The service time for all customers is supposed as 15 min and other parameters are set as shown in Table 6.

#### 5.2.2. Experimental Results

**Figure 4:**
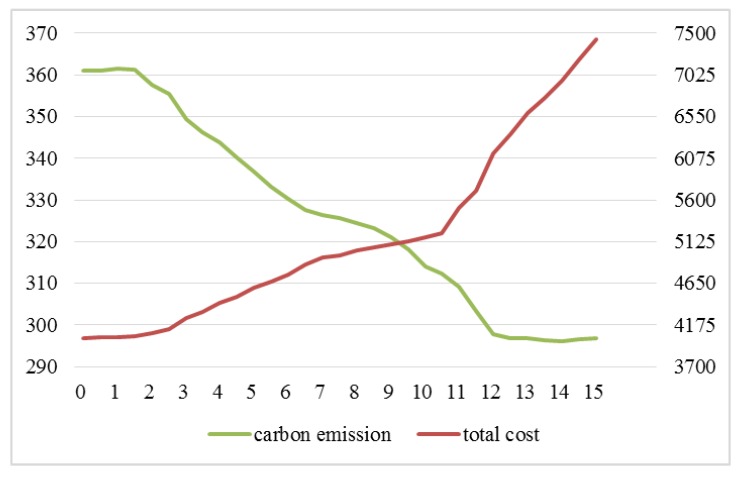
The results in consideration of carbon emission costs.

We set the value of carbon tax from 0 to 15 in the model with referring to the literature [29,36], thus getting the total cost of distribution schemes and carbon emissions, as shown in Figure 4.

From the results in Figure 4, we can observe the following findings:

(1) The total costs of the distribution scheme change with the increase of carbon tax. From Figure 4, we can see that with the increase of carbon tax, the change process of distribution scheme total costs can be divided into three stages. The first stage: when the value of carbon tax is very small (less than 1.8), the total costs increase slowly; the second stage: when the value of carbon tax is within a certain range (greater than 1.8, less than 10.7), the total costs increased moderately; the third stage: when the value of carbon tax is large (greater than 10.7), the total costs increase dramatically.
(2) Carbon emissions change with the increase of carbon tax. From Figure 4, we can see that with the increase of carbon tax, the change process of carbon emissions can be divided into three stages. The first stage: when the value of carbon tax is very small (less than 1.8), carbon emissions remain unchanged; the second stage: when the value of carbon tax is within a certain range (greater than 1.8, less than 12.1), there is continuous reduction in carbon emissions; the third stage: when the value of carbon tax is large (greater than 12.1), carbon emissions remain basically unchanged.

From Figure 4, we know the carbon emissions will not change with the different carbon tax when the carbon tax is in the range of $C_{0}\leq 1.8,C_{0}\geq 12.1$. The carbon emission decreases with the increase of $C_{0}$ when $1.8\leq C_{0}\leq 12.1$. As the result shows, the cold chain logistics enterprises can reduce the total cost of distribution by optimizing the paths when the carbon tax price gradually increases in the critical range ($1.8\leq C_{0}\leq 12.1$), and then reduce the cost pressure which is generated from the increase of carbon tax. Objectively, there are also better environmental benefits. 

**Figure 5:**
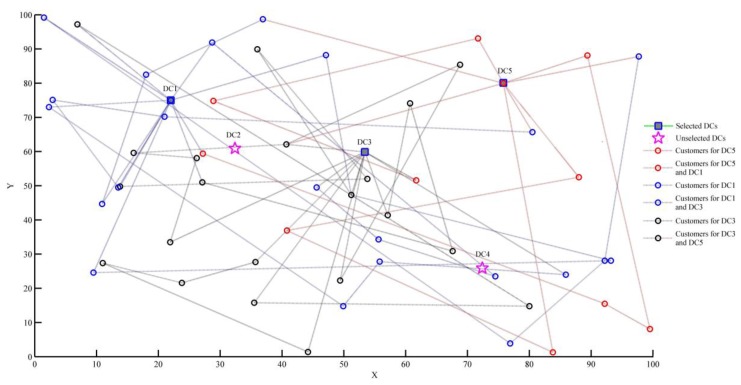
Distribution network when $C_{0}=0$.

Next, the values of carbon tax are 0 and 6, which are taken as examples to further analyze the impact of considering the carbon emission costs or not on carbon emissions and distribution schemes. As the result shows, the carbon emissions decrease with the increase of $C_{0}$ when $1.8\leq C_{0}\leq 12.1$. In the above range, we choose a carbon tax of 6 as an example to analyze. When the carbon emission costs are not considered (that is, carbon tax is 0), the obtained distribution schemes are shown in Figure 5.

The distribution task requires the joint completion of the three distribution centers DC1, DC3, and DC5, and the order of distribution services is shown in Table 7.

**Figure 6:**
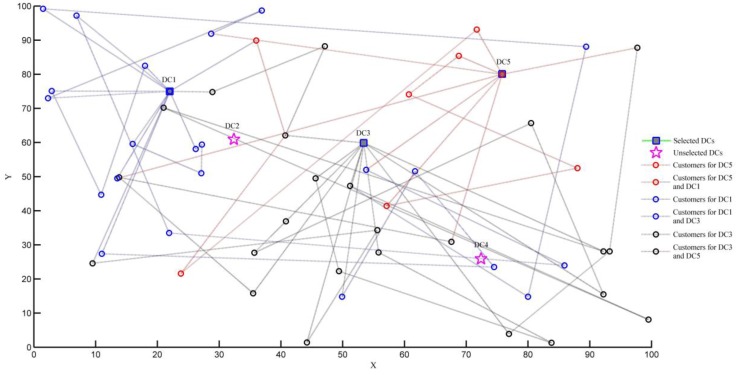
Distribution network when $C_{0}=6$.

When the carbon emission costs are considered (taking $C_{0}=6$ as example), the obtained distribution schemes are shown in Figure 6.

Similarly, the distribution task requires the joint completion of the three distribution centers DC1, DC3, and DC5, and the order of distribution services is shown in Table 8.

As shown in Table 9, the results are compared with those results obtained without considering the carbon emission costs.

From the results in Table 9, we can draw the following conclusions:

(1) There is no effect on the selection of distribution centers and the number of vehicles whether carbon emissions are considered or not. In this paper, we analyze the distribution schemes when value of carbon tax is changing in the range of 0 to 15. It was found that the selected distribution centers are DC1, DC3, and DC5 in the five candidate distribution centers, and the number of vehicles used is 15 with the change of carbon tax.
(2) The LCLRP model proposed in this paper can effectively reduce carbon emissions. As shown in Table 9, carbon emissions when considering carbon emission costs are reduced by 27.92 kg compared with ignoring carbon emissions.

#### 5.2.3. Analysis of Results

In summary, the LCLRP model proposed in this paper is mainly aimed at the combination optimization of the location–routing problem in the distribution of fresh agricultural products, and the objective function is minimizing distribution costs that include carbon emission costs to enable the logistics enterprises to achieve the unification of the economic and environmental benefits, thus “green and low-carbon” is truly realized. However, the total costs of considering carbon emissions are higher than the total costs when carbon emissions are ignored in the design of distribution path optimization of cold chain logistics, which shows that is necessary to pay a certain amount of economic costs to consider the carbon emissions and realize the green logistics in today’s increasingly harsh environment.

From the governmental agencies’ point of view, firstly, what they should do is always pay attention to environmental issues, strictly supervise the enterprises, and raise their awareness of green logistics. Secondly, the government may think about issuing a series of subsidy policies to subsidize the total costs of enterprises after considering carbon emissions (imposing carbon taxes), and encourage enterprises to take carbon emissions into consideration on their own initiative and rationally choose the route for distribution. For example, the government can carry out incentive policies for some cold chain logistics enterprises with great influence, so that they can play a leading role.

From the cold chain logistics companies’ point of view, firstly, they must take the initiative to raise awareness of environmental protection, and the carbon emission should be introduced into the distribution path optimization of cold chain logistics, so that distribution paths are rationally selected and the total costs are reduced. Secondly, consideration of carbon emissions can reduce the harm of logistics activities to the environment in the operation of logistics enterprises, which is conducive to the sustainable development of social and economic factors. Thirdly, the factor of carbon emission is taken into consideration, which promotes enterprises to fulfill their social responsibilities and establish good corporate image while in the pursuit of profit, thereby indirectly enhancing the competitiveness of enterprises.

## 6. Conclusions

As people attach importance to the global environment and as the awareness of environmental protection continues to increase, the energy-saving and emission-reduction topics have become the focus of sustainable development. In the distribution of fresh agricultural products, it is essential to optimize the design of the cold chain logistics network under the constraints of both benefit and environment. In this paper, aiming at the location–routing problem in the distribution process of fresh agricultural products, the LCLRP model is proposed and a hybrid genetic algorithm combined with heuristic rules is designed to solve the model. Finally, the simulation is carried out through a practical example to find out the results. At the same time, the impacts of carbon tax on the results (total costs and carbon emissions) are analyzed by reference to the carbon tax policies in European countries, which proves that a reasonable range of carbon tax can effectively reduce carbon emissions in cold chain logistics network. Although the carbon tax policy can promote energy conservation and emission reduction and is beneficial to the environment, it will have a negative impact on the economy in the short term. Therefore, it is required for the government and enterprises to work together to attain a balanced state of economic and environmental benefits and achieve a win-win situation. 

However, there will be a more complex environment in the actual operation of distribution. For future research, real geographical situations can be employed in the distribution path planning. For example, the introduction of a road congestion index into the model will be better able to reflect the actual feasible solution. Moreover, the uncertainty of customers’ demand may be also considered in the future research under the background of a low-carbon economy.

## Figures and Tables

**Table 1:** The meaning table of parameters and variables.

Parameters and Variables	Meaning
$L_{g}$	The collection of candidate distribution center *g*
$V_{g}$	The collection of customers assigned to distribution center *g*
$C_{0}$	The carbon tax of per unit of carbon emissions
$K_{g}$	The number of refrigerated trucks in distribution center *g*
$q_{i}$	The demand of customer *i*
$c_{ij}^{k}$	The transportation costs for unit distance when refrigerated truck from customer *i* to customer *j*
$C_{k}$	Fixed cost of refrigerated truck *k*
$d_{ij}$	The distance between customer *i* and customer *j*
$Q_{k}$	The maximum load allowed for refrigerated truck
$Q_{ij}^{k}$	The weight of the loaded cargoes when the refrigerated truck travels between customer *i* and customer *j*
$t_{si}$	The time required for refrigerated trucks to serve customer *i*
$Q_{g}$	The maximum capacity of the distribution center *g*
$t_{ij}^{k}$	The travel time of refrigerated truck *k* from customer *i* to customer *j*
$t_{i}^{k}$	The time to arrive at the customer *i* for refrigerated truck *k*
$x_{ij}^{k}$	$x_{ij}^{k}=1$ represents the refrigerated truck passes through the road between customer *i* and customer *j*, otherwise $x_{ij}^{k}=0$
∂	The spoilage rate of cargoes in refrigerated truck
$Z_{g}$	$Z_{g}=1$ represents distribution center *g* is opened, otherwise $Z_{g}=0$
P	Unit value of cargoes in refrigerated truck
$C_{e}$	The refrigeration costs for unit time during transportation process of refrigerated trucks
$C_{e}^{\prime }$	The refrigeration costs for unit time during unloading process of refrigerated trucks
$w_{i}$	The unloading time for refrigerated truck *k* serves the customer *i*
$C_{g}$	Fixed costs of distribution center *g*
$\mu_{1}$	The waiting costs for unit time when the refrigerated truck arrives at customer node in advance
$\mu_{2}$	The penalty costs for unit time when the refrigerated truck is late to customer node
$\left[ET_{i},LT_{i}\right]$	Time window required by customer *i* (namely, the range of time)
ρ	The fuel consumption of refrigerated truck for unit distance
ω	Carbon emissions generated from refrigeration equipment when distributing unit weight cargoes during driving unit distance
$\rho_{0}$	The fuel consumption for unit distance when refrigerated truck is empty
$EM_{g}$	Fixed carbon emissions of candidate distribution center *g*
$e_{0}$	Carbon emissions generated by unit fuel consumption
$\rho^{*}$	The fuel consumption for unit distance when refrigerated truck is fully loaded
$y_{i}^{k}$	$y_{i}^{k}=1$ represents the refrigerated truck *k* serves the customer *i*, otherwise $y_{i}^{k}=0$
t	Transportation time of cargoes in refrigerated truck
$Y_{k}$	$Y_{k}=1$ represents refrigerated truck *k* is used in distribution center, otherwise $Y_{k}=0$
$y_{ig}$	$y_{ig}=1$ represents the customer *i* is serviced by the refrigerated truck of distribution center *g*, otherwise $y_{ig}=0$
*v*	The speed of refrigerated truck

**Table 2:** Experimental results of HGA and other algorithms.

Examples				GA			CEGA			HGA	
*n*	*m*	Type		Cost	*t*		Cost	*t*		Cost	*t*
20	5	1a		55,021.32	0.20		54,986.19	0.20		54,879.53	0.20
20	5	1b		39,213.41	0.20		39,236.57	0.20		39,135.17	0.20
50	5	2a		88,795.58	3.30		89,016.14	2.50		88,681.29	2.00
50	5	2b		68,153.46	3.00		69,275.91	2.50		67,850.34	2.00
100	5	3a		204,489.12	24.60		203,985.20	23.40		203,568.61	19.50
100	5	3b		154,798.25	20.70		154,157.38	36.00		153,952.43	25.80
100	10	2a		257,932.23	38.60		247,073.29	40.90		248,965.37	29.40
100	10	2b		206,983.27	35.80		206,879.46	32.50		206,139.54	31.80
200	10	1a		498,960.38	456.80		481,283.24	412.30		483,073.98	349.20
200	10	1b		416,089.37	419.70		408,937.25	586.10		398,956.18	338.50

GA, genetic algorithm; CEGA, cycle evolutionary genetic algorithm; HGA, hybrid genetic algorithm.

**Table 3:** The location of candidate distribution center.

Serial Number	x Coordinate	y Coordinate
1	22.0	75.0
2	32.4	60.9
3	53.4	59.9
4	72.4	25.9
5	75.8	80.1

**Table 4:** Demand information of customers.

Demand Point	x Coordinate	Y Coordinate	Urgently Needed Goods 1	Ordinary Goods 1	Urgently Needed Goods 2	Ordinary Goods 2	Service Demand Time	
1	80.5	65.7	6	19	25	34	1.3	1.8
2	13.8	49.8	10	31	30	45	1.5	2
3	21	70.2	3	8	28	52	2	2.5
4	60.7	74.1	10	30	27	31	0.5	1
5	26.2	58.1	21	33	23	42	1.4	1.9
6	28.9	74.8	2	7	32	35	2.5	3
7	1.5	99.2	13	21	37	46	2	2.5
8	51.2	47.3	2	5	35	58	0.4	0.9
9	45.6	49.5	5	15	36	35	2	2.5
10	58.8	85.4	1	3	34	46	1.3	1.8
11	35.7	27.7	3	8	25	42	2.2	2.7
12	13.5	49.5	16	38	45	45	2.1	2.6
13	40.8	36.9	34	40	65	100	2.5	3
14	28.7	91.9	29	49	28	65	2.1	2.6
15	80	14.8	3	10	33	75	2.5	3
16	40.7	62.1	10	30	42	52	1.3	1.8
17	2.3	73	5	15	32	60	2	2.5
18	76.9	3.9	1	3	31	52	2.5	3
19	53.8	52	3	9	22	60	2.1	2.6
20	10.9	44.7	5	16	40	60	2	2.5
21	61.7	51.6	9	26	25	48	2.2	2.7
22	44.2	1.4	16	48	46	65	1.3	1.8
23	88	52.5	4	11	25	52	1.3	1.8
24	92.2	28.1	8	9	28	55	2.1	2.6
25	23.8	21.6	9	19	29	42	2.3	2.8
26	67.6	30.9	5	10	26	62	2.3	2.8
27	11	27.4	11	16	32	53	2.3	2.8
28	57.1	41.4	7	24	24	66	1.3	1.8
29	21.9	33.5	10	31	31	48	2.5	3
30	2.9	75.1	13	27	32	65	2.5	3
31	93.2	28.1	8	13	28	40	2.5	3
32	49.9	14.8	6	15	32	45	2.3	2.8
33	36.9	98.7	9	12	35	64	2	2.5
34	9.8	66.3	7	21	22	25	2.3	2.8
35	18	82.5	14	12	30	46	2.3	2.8
36	74.5	23.5	9	15	25	42	2.5	3
37	36	89.9	6	8	24	50	2.5	3
38	6.9	97.2	11	13	25	46	2.3	2.8
39	89.4	88.1	7	14	28	38	2.5	3
40	68.8	85.4	10	18	32	35	1.3	1.8
41	16	59.6	13	20	24	65	2	2.5
42	97.7	87.8	9	13	35	62	2.1	2.6
43	55.8	27.8	10	14	25	55	2.1	2.6
44	40.7	62.1	5	7	32	44	2.5	3
45	85.9	24	3	20	32	55	2.5	3
46	27.1	51	12	13	42	85	2.3	2.8
47	71.7	93.1	6	30	62	115	1.3	1.8
48	35.5	15.8	8	16	45	45	2.2	2.7
49	19.4	18	11	19	35	62	2.2	2.7
50	92.2	15.5	4	6	33	65	2.1	2.6
51	45.6	49.5	7	8	28	48	2.1	2.6
52	99.5	8.1	9	12	25	52	2.5	3
53	55.6	34.3	18	20	47	52	2.3	2.8
54	83.8	1.3	2	9	25	64	2.3	2.8
55	51.2	47.3	16	19	28	41	2	2.5
56	47.1	88.2	15	22	24	38	2.1	2.6
57	49.4	22.3	21	21	39	45	2.5	3
58	56.7	92.2	15	18	25	44	1.3	1.8
59	9.5	24.6	8	17	36	45	1.7	2.2
60	27.2	59.4	19	26	24	38	1	1.5

**Table 5:** Vehicle parameters.

Parameter	Parameter Value	Parameter	Parameter Value
Outline dimension (mm)	4955 × 1720 × 2455	Container size (mm)	2795 × 1545 × 1535
Total mass (kg)	1620	Rated load capacity (kg)	795
Engine type	LJ469Q-1AE9	Maximum speed (km/h)	120
Fuel type	gasoline	Integrated fuelconsumption	8.8 L/100km
Swept volume(mL)	1249	Engine power (kw)	64

**Table 6:** Model parameter settings.

Parameter	Parameter Value
*P*	10 Yuan/kg
$\partial_{1}$	0.002
$\partial_{2}$	0.003
$C_{e}$	15 Yuan/h
$C_{e}^{\prime }$	20 Yuan/h
$\mu_{1}$	300 Yuan/h
$\mu_{2}$	300 Yuan/h
τ	100 Yuan/t
$\rho^{*}$	0.377 L/km
$\rho_{0}$	0.165 L/km
$e_{0}$	2.63 kg/L
ω	0.0066 g/kg·km
ν	30 km/h

**Table 7:** The distribution schemes when $C_{0}=0$.

Route Number	Distribution Service Order	Route Number	Distribution Service Order
1	DC5-42-24-18-7-DC1	9	DC3-44-40-57-DC3
2	DC3-51-53-36-14-DC1	10	DC1-38-15-48-DC3
3	DC3-45-43-32-17-DC1	11	DC1-60-50-52-39-DC5
4	DC1-56-55-31-59-DC1	12	DC1-46-26- 4-28-DC3
5	DC3-21-6-47-DC5	13	DC5-33-35-20-DC1
6	DC3-11-25-27-22-DC3	14	DC5-54-13-23-DC5
7	DC3-16-41-5-29-DC3	15	DC5-1-3-30-12-DC1
8	DC1-2-19-37-8-DC3		

**Table 8:** The distribution schemes when $C_{0}=6$.

Route Number	Distribution Service Order	Route Number	Distribution Service Order
1	DC3-55-15-39-7-DC1	9	DC3-50-1-11-13-DC3
2	DC3-32-21-36-27-DC1	10	DC5-26-2-12-48-DC3
3	DC1-35-20-30-DC1	11	DC1-6-56-16-DC3
4	DC3-43-54-57-51-DC3	12	DC5-42-31-18-DC3
5	DC3-24-3-52-8-DC3	13	DC5-14-33-17-DC1
6	DC1-59-53-22-DC3	14	DC5-28-23-4-40-DC5
7	DC1-37-44-25-47-DC5	15	DC5-19-45-29-38-DC1
8	DC1-5-60-46-41-DC1		

**Table 9:** Results comparison when $C_{0}=0$ and $C_{0}=6$.

$C_{0}$	Distribution Center	The Number of Vehicles	Total Casts (¥)	Carbon Emissions (kg)
0	DC1\DC3\DC5	15	3827.03	361.04
6	DC1\DC3\DC5	15	4671.83	333.12
